# Supplementary material for: Complex‐centric proteome profiling by SEC‐SWATH‐MS
Source: Mol Syst Biol. 2019 Jan 14;15(1):e8438. doi: 10.15252/msb.20188438 (PMC6346213; doi:10.15252/msb.20188438)
Supplement: Supplementary file 7 — Dataset EV6 [file MSB-15-e8438-s007.zip › feature_plots_bioplex/Q9NV96.pdf]

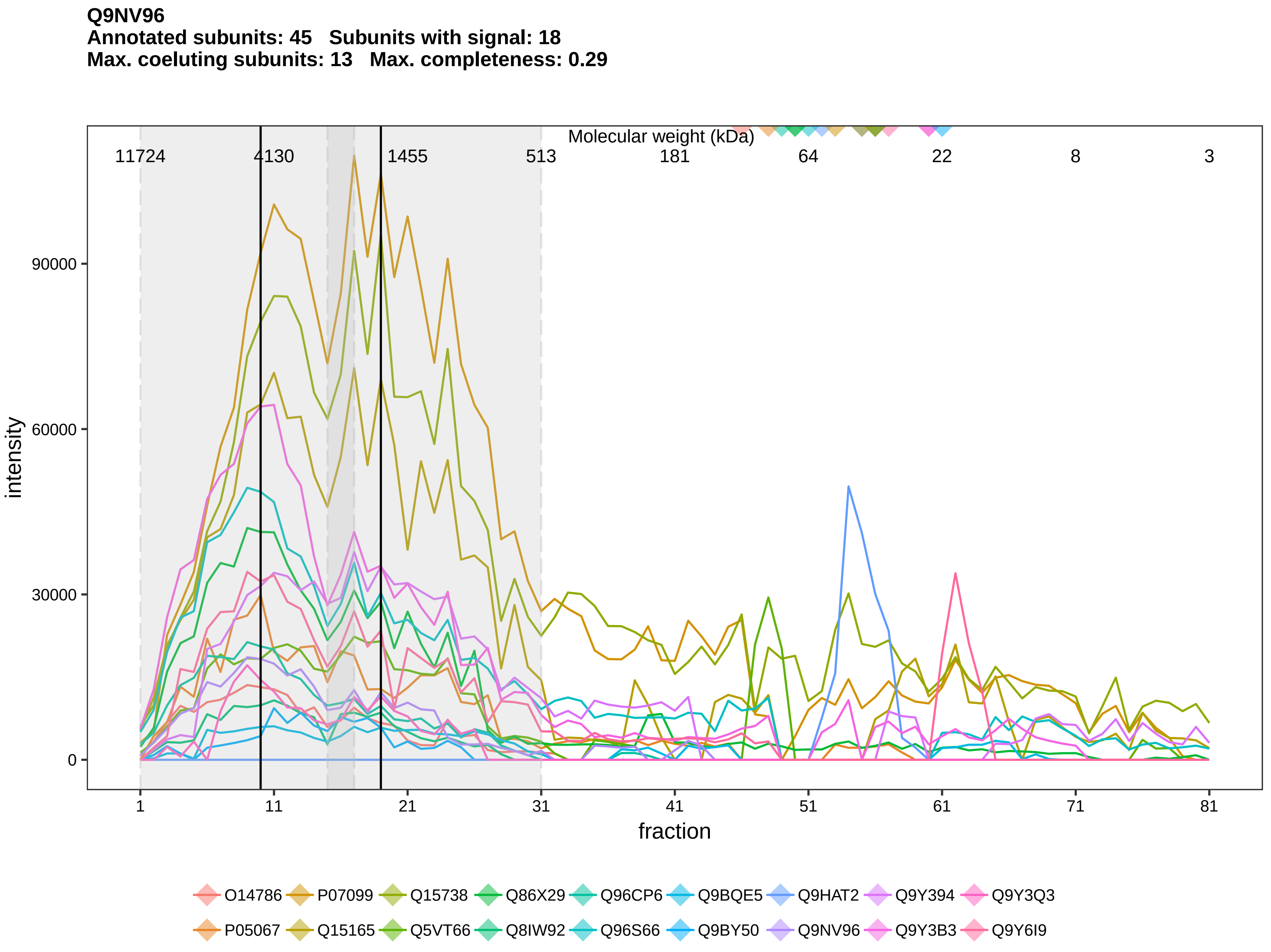
Q9NV96
Annotated subunits: 45 Subunits with signal: 18
Max. coeluting subunits: 13 Max. completeness: 0.29
Molecular weight (kDa)
11724
4130
1455
513
181
64
22
8
3
intensity
90000
60000
30000
0
1
11
21
31
41
51
61
71
81
fraction
O14786
P07099
Q15738
Q86X29
Q96CP6
Q9BQE5
Q9HAT2
Q9Y394
Q9Y3Q3
P05067
Q15165
Q5VT66
Q8IW92
Q96S66
Q9BY50
Q9NV96
Q9Y3B3
Q9Y6I9
